# Myopathy-inducing mutation H40Y in *ACTA1* hampers actin filament structure and function

**DOI:** 10.1016/j.bbadis.2016.04.013

**Published:** 2016-08

**Authors:** Chun Chan, Jun Fan, Andrew E. Messer, Steve B. Marston, Hiroyuki Iwamoto, Julien Ochala

**Affiliations:** a Department of Physics and Materials Science, City University of Hong Kong, Hong Kong; b City University of Hong Kong Shenzhen Research Institute, Shenzhen, China; c National Heart and Lung Institute, Imperial College London, London, UK; d Japan Synchrotron Radiation Research Institute, SPring8, Hyogo, Japan; e Centre of Human and Aerospace Physiological Sciences, King's College London, London, UK

**Keywords:** Myopathy, Actin, Contractile dysfunction, Small-angle X-ray scattering, In vitro motility assay, Molecular dynamics

## Abstract

In humans, more than 200 missense mutations have been identified in the *ACTA1* gene. The exact molecular mechanisms by which, these particular mutations become toxic and lead to muscle weakness and myopathies remain obscure. To address this, here, we performed a molecular dynamics simulation, and we used a broad range of biophysical assays to determine how the lethal and myopathy-related H40Y amino acid substitution in actin affects the structure, stability, and function of this protein. Interestingly, our results showed that H40Y severely disrupts the DNase I-binding-loop structure and actin filaments. In addition, we observed that normal and mutant actin monomers are likely to form distinctive homopolymers, with mutant filaments being very stiff, and not supporting proper myosin binding. These phenomena underlie the toxicity of H40Y and may be considered as important triggering factors for the contractile dysfunction, muscle weakness and disease phenotype seen in patients.

## Highlights

• H40Y stiffens the entire actin filament.
• H40Y partially limits actin filament extensibility upon activation.
• H40Y disrupts myosin and tropomyosin function.

## 1 Introduction

More than 200 mutations in the *ACTA1* gene have been observed and these are usually associated with life-threatening diseases termed congenital myopathies. One common pathological feature for most of the patients is severe weakness in their limb, masticatory and respiratory muscles [1], [2]. Little is known about the underlying biochemical and biophysical mechanisms by which defects in skeletal muscle α-actin ultimately lead to muscle weakness [1], [2]. Hence, no cure exists; treatment simply focuses on symptomatic management such as respiratory intervention, particularly nocturnal ventilation [1], [2].

In skeletal muscle, actin monomers are crucial for force production and contraction as they directly interact with tropomyosin and myosin. In the absence of Ca^2 +^, tropomyosin sterically hinders interactions between actin and myosin molecules. Upon addition of Ca^2 +^, tropomyosin moves over the surface of actin exposing myosin-binding sites on actin filaments. Myosin can then bind to actin monomers and form myosin cross-bridges, allowing the production of force and motion [3]. Most *ACTA1* mutations are missense mutations leading to the substitution of just one residue in the skeletal muscle α-actin protein. Interestingly, we have shown that, in the presence of these amino acid substitutions such as the lethal H40Y [4], the myosin binding to actin filaments is greatly disrupted limiting the intrinsic force-generating capacity [5]. In the present study, by using a unique combination of molecular dynamics simulation, small-angle X-ray scattering and in vitro motility assay, we aimed to identify the mechanisms by which the myosin attachment to actin monomers is partially prevented in the presence of H40Y.

H40Y is located in actin sub-domain 2, in the DNase I-binding loop (also called D-loop, residues 38–52). The D-loop establishes a lock-and-key interaction with the neighbouring actin monomer by interacting with the C terminus of the adjacent subunit in actin filament. The interface is large and is stabilized by electrostatic and hydrophobic interactions and also by geometric surface complementarity. Hence, we initially hypothesised that the inefficient myosin binding would originate from an altered D-loop structure and actin–actin interface. This would, upon activation, prevent changes in the twist of the F-actin helix, limiting filament extension and reducing the axial range for myosin heads to find target zones along the actin filaments. As a consequence, it would provide less binding sites for myosin molecules.

## 2 Materials and methods

### 2.1 Animals

Two- to three-month old male mice expressing the H40Y skeletal muscle α-actin mutant were analysed along with wild-type strain-, age- and gender-matched mice. For a complete description of the knock-in mice, please see [4], [5]. Mice were killed by cervical dislocation under deep isoflurane sedation and skeletal muscles (Tibialis anterior, TA; and extensor digitorum longus, EDL) were dissected. All procedures involving animal care, welfare and handling were performed according to institutional guidelines and were reviewed and approved by the Animal Ethics Committee of Uppsala University.

### 2.2 Muscle preparation and myofibre permeabilization

TA and EDL muscles were separated into two portions. One portion was frozen in liquid nitrogen-chilled propane and stored at − 80 °C. The other portion was placed in relaxing solution at 4 °C. Bundles of approximately 50 myofibres were dissected free and then tied with surgical silk to glass capillary tubes at slightly stretched lengths. They were then treated with skinning solution (relaxing solution containing glycerol; 50:50 v/v) for 24 h at 4 °C, after which they were transferred to − 20 °C. In addition, the muscle bundles were treated with sucrose, a cryoprotectant, within 1–2 weeks for long-term storage (17). They were detached from the capillary tubes and snap frozen in liquid nitrogen-chilled propane and stored at − 80 °C.

### 2.3 X-ray diffraction recordings and analyses

Two to three days prior to X-ray recordings, bundles were de-sucrosed, transferred to a relaxing solution and single myofibres were dissected. Arrays of approximately 30 myofibres were set up (18–23). For each myofibre, both ends were clamped to half-split gold meshes for electron microscopy (width = 3 mm), which had been glued to precision-machined ceramic chips (width = 3 mm) designed to fit to a specimen chamber. The arrays were then transferred to the skinning solution and stored at − 20 °C. Approximately 80 arrays were mounted (10 arrays per mouse – four knock-in and four wild-type mice – corresponding to approximately 2400 attached fibres).

On the day of X-ray recordings, arrays were placed in a plastic dish containing a pre-activating solution and washed thoroughly to remove the glycerol. They were then transferred to the specimen chamber, capable of manual length adjustment and force measurement (force transducer, AE801, Memscap, Bernin, France), filled with a pre-activating solution. Mean sarcomere length was measured and set to 2.50 μm or > 3.60 μm. Subsequently, for arrays at a sarcomere length equal to 2.50 μm, X-ray diffraction patterns were recorded at 15 °C, first in the pre-activating solution and then in the activating solution (pCa 4.5) when maximal steady-state isometric force was reached. It should be mentioned that the activating solution was supplied to the chamber by using a remote-controlled pump. For arrays at a sarcomere length > 3.60 μm, the protocol was identical except that pre-activating and activating solutions were replaced by low-EGTA rigor and calcium-rigor solutions with 2,3-butanedione monoxime (to prevent major sarcomere in-homogeneities).

For each array, approximately 20 to 30 diffraction patterns were recorded (depending on myofibre length) for each solution at the BL45XU beamline of SPring-8. The wavelength was 0.1 nm, and the specimen-to-detector distance was 2.00 m or 3.47 m (to maximize the spatial resolution to determine filament compliance). As a detector, a cooled CCD camera (C4880, Hamamatsu Photonics; 1000 × 1018 pixels) was used in combination with an image intensifier (VP5445, Hamamatsu Photonics). To minimize radiation damage, the exposure time was kept low (1–2 s) and the specimen chamber was moved by 100 μm after each exposure. Moreover, we placed an aluminium plate (thickness, 0.35–0.5 mm) upstream of the specimen chamber. Following the X-ray recordings, background scattering was subtracted, and reflection intensities were determined as described elsewhere [6], [7], [8], [9], [10].

Relaxing and activating solutions contained 4 mM Mg-ATP, 1 mM free Mg^2 +^, 20 mM imidazole, 7 mM EGTA, 14.5 mM creatine phosphate, 324 U/mL creatine phosphokinase, 1000 U/mL catalase, and KCl to adjust the ionic strength to 180 mM and pH to 7.0*.* Dithiothreitol (DTT) was also added (10 mM). The pre-activating solution was identical to the relaxing solution, except that the EGTA concentration was reduced to 0.5 mM. The concentrations of free Ca^2 +^ were 10^–9.0^ M (relaxing and pre-activating solutions, pCa 9.0) and 10^–4.5^ M (activating solution, pCa 4.5).

### 2.4 In vitro motility assay

Skeletal muscle α-actin was purified from thin filaments isolated from frozen wild-type and knock-in mouse leg muscles by the method based on that described in Song and co-workers and labelled with TRITC phalloidin [11]. The movement of individual actin filaments (more than 100 per condition) over a bed of immobilized rabbit fast skeletal muscle heavy meromyosin (100 μg/mL) was then evaluated in a motility chamber as previously described [12]. Filament movement was recorded and manually tracked yielding speed data [13], [14].

### 2.5 Molecular dynamics simulation

One actin filament containing the H40Y mutants composed of subunits with bound Mg^2 +^–ADP was constructed. This was based on vertebrate (rabbit) skeletal muscle actin Protein Data Bank entry 2ZWH, with a high-affinity Mg^2 +^ cation placed at the nucleotide-binding site and the first solvation shell of explicit waters included. The filament contained 13-monomer subunits as described in [15], [16], [17]. The filament was solvated in explicit TIP3P water molecules, with K^+^ and Cl^−^ ions included at a final concentration of 0.18 M using the solvate and autoionized tools in VMD [18]. Periodic boundary conditions were introduced such that the filament was aligned to repeat along the z direction, interacting with itself at the box edges.

The MD simulations were performed using NAMD 2.10 package [19] and the CHARMM 22/27 force field [20] with CMAP correction [21]. Electrostatic interactions were calculated using the particle mesh Ewald sum method with a cutoff of 12 Å. All hydrogen-containing covalent bonds were constrained by the SHAKE algorithm, therefore allowing an integration time step of 2 fs. Before production runs, an actin filament system was energy minimized, heated, and pre-equilibrated for 100 ps in the canonical ensemble whilst the protein backbone, the nucleotide, the active site Mg^2 +^, and water oxygen atoms were harmonically restrained with spring constant 1 kcal/mol Å^2^. Simulations were then continued in the constant NPT ensemble (310 K and 1 atm) for an additional 200 ps. Langevin thermostats with a damping coefficient of 0.5 ps^− 1^ were used to control the system temperature. A Langevin-piston barostat with a piston period of 2 ps and a damping time of 2 ps was used to control the pressure. Constraints were next released step-wise (with spring constant gradually decreasing from 1 kcal/mol Å^2^ to 0 by steps of 0.1 kcal/mol Å^2^) over a total of 100 ps before starting the production runs. A total of 245 ns of data were generated for the H40Y actin filament systems. Only data after the system were equilibrated were taken for further analysis. The final 190 ns of data were used for analysis unless otherwise specified. All quantities presented in this article are averaged value over all subunits and the simulation windows. All MD data are analysed based on the block averages method [22]. All errors in the MD results section refer to standard deviation (SD).

### 2.6 Statistics

The unpaired Student's t-test was applied, and in cases where the data did not meet the criteria of normality (Kolmogorov–Smirnov test, *p* < 0.05), the non-parametric Mann–Whitney rank-sum test was performed.

## 3 Results and discussion

In the present study, we unravelled for the first time the molecular negative events by which the toxic H40Y limits the myosin attachment to actin monomers, and reduces the proportion of strongly bound myosin cross-bridges. For that, we started by performing a molecular dynamics simulation, we then ran a broad range of biophysical assays including small-angle X-ray scattering and in vitro motility assay on muscles from one knock-in mouse model perfectly recapitulating the human condition [4], [5].

### 3.1 H40Y stiffens the entire actin filament

The persistence length of actin filament containing H40Y was evaluated using MD simulation. It was equal to 12.21 ± 4.64 μm, which is larger than the value previously reported for wild-type (WT) filament (9.8 ± 0.14 μm [23]) with *p* < 0.018. This demonstrates that H40Y stiffens actin filaments. To understand the potential underlying mechanisms, we measured the crossover length of the filament with thirteen H40Y subunits. The crossover length was 357.43 ± 0.60 Å, which is smaller than that of WT filament (365 ± 15 Å [24]) with *p* < 0.00003. In addition, the number of longitudinal contacts between the H40Y D-loop and the subunit located above it along the same strand became larger compared to that of WT filament. Specifically, all contacts increased between residues 37-52 (D-loop) and between residues 130-150, 161–175, 345–357, and 369–375 of the above subunit. All the equilibrated distances between representative structures and coarse-grained (CG) sub-groups of the different subunits are summarized in Table 1. All the longitudinal and lateral contact distances were found to decrease due to H40Y, except for residue 61–residue 169 (D-loop versus SD1) which increased by a small amount and residue 205–residue 286 and residue 241–residue 322 (SG4 versus SG3) which were very close to the WT value. Most of the decreases in equilibrated distances were associated to D-loop structure and explained the more frequent contacts between D-loop and the above subunits. These data suggest that in the presence of H40Y, the actin subunits become closer, resulting in an unexpectedly large amount of longitudinal interactions, stiffening the filament.

We further examined the subunit geometries through coarse-graining residues into relatively rigid sub-groups: residues 5–33, 80–147, and 334–349 as SG1; residues 34–39 and 52–59 as SG2; residues 148–179 and 273–333 as SG3; residues 180–219 and 252–262 as SG4. We denoted the centres of geometry (COGs) for these residues as R1, R2, R3, and R4. Concerning equilibrated structural parameters of filament subunits (inter-subunit bond distances, angles, and dihedral angles; Table 2), a few major differences emerged between H40Y and WT filament subunits. The equilibrated R1–R2 bond distances differed by a considerate amount (21.62 ± 0.35 and 22.99 ± 0.39 for H40Y and WT, respectively). This bond distance characterizes the extended distance of SG2 from crystal structure of WT actin. Further, the equilibrated R2–R1–R3 angles increased significantly from 102.06 ± 2.06 to 105.35 ± 1.90 after incorporating the mutation. Also, the equilibrated R2–R1–R3–R4 dihedral angles slightly increased from 10.27 ± 3.41 to 11.14 ± 2.61. All these data suggest that H40Y alters the subunit structure by shifting the position of SG2 and thus introduces new interactions associated to the D-loop with adjacent subunits. In this way, H40Y strengthens the actin filament through intra-subunit interactions. To conclude, the stiffness of H40Y actin filament originates from a dramatic strengthening of both longitudinal and lateral subunit–D-loop contacts.

### 3.2 H40Y partially limits actin filament extensibility upon activation

**Fig. 1:**
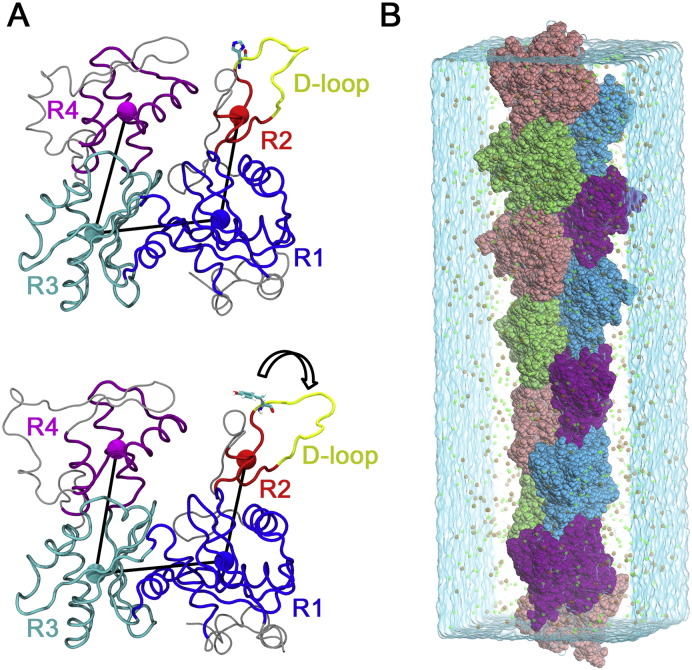
Molecular dynamics simulation setup. (A) Actin subunit structure with rigid portions of SG1, SG2, SG3, and SG4 shown in blue, red, cyan, and magenta, respectively, and the COG of each rigid group is represented in a sphere of the same colour, labelled as R1, R2, R3, and R4. Upper panel is the subunit crystal structure (PDB ID: 2ZWH) and the lower panel is the equilibrated H40Y structure after molecular dynamics simulation. Subunit structure D-loop is shown and labelled in yellow. (B) An effective infinitely long H40Y actin filament in a periodic boundary condition (PBC) water box under physiological conditions.

Simulations tend to suggest that actin filament compliance or extensibility modulates the formation of the myosin cross-bridges and force production [25], [26], [27]. Indeed, actin filaments slightly change their helical symmetry and reach their most untwisted and longest length when activated [27], [28]. When extended, F-actin expands the axial range for myosin heads to find target zones along the actin filaments, and as a consequence, provides more binding sites for myosin molecules when compared with rigid actin filaments [26]. (Fig. 1).

**Fig. 2:**
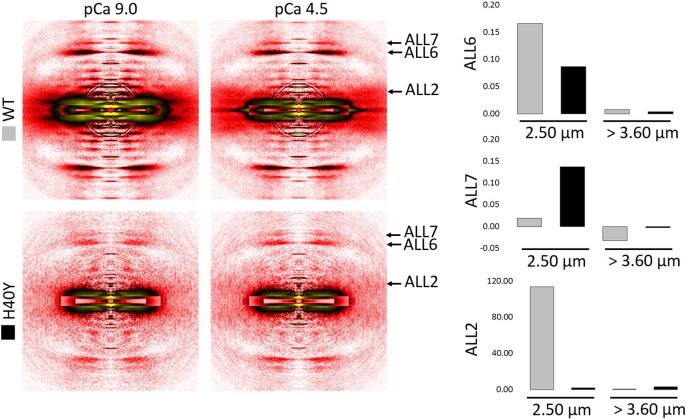
Actin-layer line (ALL) spacing and intensity profiles. X-ray diffraction patterns (specimen-to-detector distance: 2.50 m) of membrane-permeabilised myofibres set at a sarcomere length of 2.50 μm from WT (first row) and from H40Y (second row) mice in resting (pCa 9.0, first column) and maximal activating (pCa 4.5, second column) conditions. The ALL6 and ALL7 actin spacing increments (in %) as well as the ALL2 intensity increment (in fold-change) during activation are presented. Five arrays of approximately 30 fibres were tested per mouse (four knock-in and four wild-type mice) for these experiments.

To experimentally evaluate F-actin extensibility in the presence of H40Y, we recorded the X-ray diffraction patterns of relaxed and activated single membrane-permeabilized myofibres from WT mice and from knock-in rodents expressing H40Y. X-ray experiments were possible as the overall sarcomere ultrastructure is preserved in the presence of H40Y [5]. We specifically evaluated the actin spacing, which represents the axial distance between actin monomers averaged over the whole thin filament in sarcomeres [27]. The determination of actin spacing (or actin extensibility) is often achieved using actin layer line (ALL) reflections, i.e., 6th ALL (ALL6, d = 5.9 nm) and 7th ALL (ALL7, d = 5.1 nm) [27]. In the present study, at an optimal thick and thin filament overlap (mean sarcomere length of 2.50–2.60 μm), the peaks of ALL6 and ALL7 shifted upon activation. These shifts differed between WT and H40Y myofibres but in opposite directions (Fig. 2). The ALL6 peak shift was smaller for H40Y myofibres when compared with WT cells (~ 0.08% versus ~ 0.17%) whilst the ALL7 peak shift was greater (~ 0.13% versus ~ 0.02%). Hence, the results are difficult to interpret. Estimating the actin spacing from ALL6 and ALL7 often leads to errors as an imperfect alignment of filament axis may result in the arching of these particular reflections. This arching is hardly recognizable by the naked eye as the extension of F-actin during contraction is less than 1% (Fig. 2). In addition, the peaks of ALL6 and ALL7 are broad, leading to additional errors. ALL7 was even weaker than ALL6, increasing the probability of errors or misinterpretation. Hence, determining the actin spacing using other reflections such as meridional reflections (known to be less influenced by imperfect alignments) may overcome such limitations. Following this, we originally recorded and analysed the 3rd order meridional reflection of troponin (TN3, d = 12.9 nm) to calculate the actin spacing. Its intensity was stably observed in the X-ray diffraction patterns of membrane-permeabilized myofibres.

**Fig. 3:**
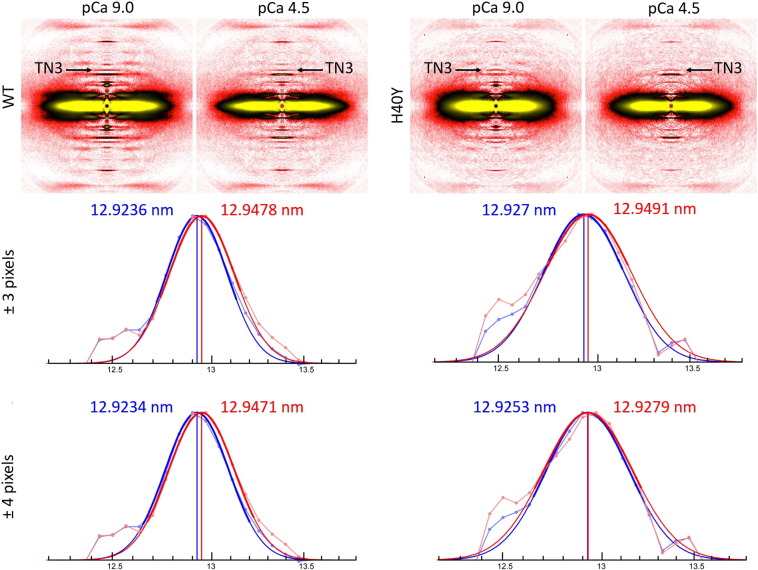
TN3 intensity profiles. X-ray diffraction patterns (specimen-to-detector distance: 3.47 m) of membrane-permeabilised myofibres set at a sarcomere length of 2.50 μm from WT (first column) and from H40Y (second column) mice in resting (pCa 9.0, blue lines on graphs) and maximal activating (pCa 4.5, red lines on graphs) conditions. The thinner red and blue curves represent the observed data, and the thicker red and blue curves represent the fitted Gaussian function with a range of fitting ± 4 pixels or ± 3 pixels. For these experiments, five arrays of approximately 30 fibres were mounted per mouse (four knock-in and four wild-type mice).

After subtracting the background scattering [6], [7], [8], [9], [10], the intensity profile of TN3 was fitted to the Gaussian function, and its centre was regarded as the position of the peak. Applying this method resulted in a sub-pixel resolution (Fig. 3). For fitting purposes, data in the ranges of ± 4 as well as ± 3 pixels from the observed peak were used. In WT myofibres, at an optimal thick and thin filament overlap, the peak shifted by ~ 0.18% upon maximal activation. This is in accordance with our ALL6 results and with data from the literature which observed an increase of ~ 0.20% during maximum tension, corresponding to an elongation of ~ 3 nm for the actin filaments per half-sarcomere [27]. Interestingly, in H40Y myofibres, the widths of the observed peaks were visibly wider (standard deviation: 9–10 versus 5–6 pixels). Also the peak became asymmetric after maximal activation. For data in the range of ± 4 pixels, the peak position changed by only ~ 0.02% between pCa 9.0 and pCa 4.5 whilst for data in the narrower range of ± 3 pixels, it shifted by ~ 0.17%, i.e., close to the value obtained for WT myofibres. These observations suggest that normal and mutant actin molecules in knock-in animals behave differently. The wider TN3 peaks, observed even in myofibres at pCa 9.0 (rest), suggest that the mutant actin repeat is intrinsically more variable. If normal and mutant actin monomers were forming uniform copolymers, then there would not be any asymmetry during contraction. Asymmetry has to be caused by normal and mutant actin molecules being segregated in distinctive homopolymers. This implies that actin monomers containing H40Y are intrinsically polymerized in distinct filaments when compared with normal WT actin monomers.

H40Y may primarily stiffen actin monomers and partially prevent filament untwisting upon activation. Considering that H40Y reduces the cross-bridge number and force-generating capacity by ~ 50% [5], and that the amount of mutant proteins in myofibres is ~ 40% [4], one would suggest that stiff actin mutant homopolymers do not support the production of force at all.

### 3.3 H40Y disrupts the contractile function by perturbing interactions with myosin and indirectly with tropomyosin

**Fig. 4:**
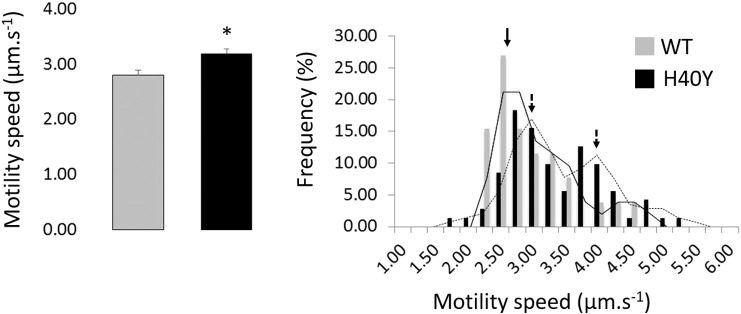
Actin sliding speed. This figure displays the means/standard errors as well as distribution of speeds for individual filaments coming from WT and H40Y mouse muscles. The star denotes a significant difference between WT and H40Y (*p* < 0.05).

We purified actin from muscles of WT animals and of knock-in mice carrying H40Y. This step was possible as H40Y does not affect actin polymerization rate [29], even though the D-loop is thought to play a role in actin dynamics by slightly modifying its conformation [30]. An unloaded in vitro motility assay was then used to measure the speed (V_f_) at which myosin molecules move WT versus H40Y actin filaments. We observed a Gaussian distribution for WT filaments but noticed a biphasic distribution for filaments coming from muscles carrying H40Y, with one sub-population behaving as usual (normal V_f_, 58% of the filaments had a velocity < 3.50 μm·s^− 1^) and another being abnormal (increased V_f_, 42% of the filaments had a velocity > 3.50 μm·s^− 1^) (Fig. 4). This finding strengthens our suggestion that normal and mutant actin monomers are likely to form distinctive homopolymers.

The increased speed of myosin-powered movement for H40Y filaments may arise from a change in the properties of the actin filament per se. As previously suggested using a computational approach [31], the stiffening of the mutant actin filaments may increase the cycling of individual myosin heads by shortening the time spent by individual myosin molecules in the strong binding state.

In addition to modifying myosin function, we suspected that H40Y would also alter tropomyosin movement. To verify this, we used single membrane-permeabilized myofibres from WT rodents and from knock-in mice expressing H40Y. We then monitored the far off-meridional part of the 2nd ALL (ALL2, d = 19 nm) and measured its intensity change during activation under various conditions. ALL2 is one of the most documented reflections in vertebrate muscle. During activation, its intensification originates from movement of tropomyosin over the thin filament, exposing myosin binding sites on actin filaments [32]. Here, at an optimal thick and thin filament overlap (mean sarcomere length of 2.50–2.60 μm), we observed a weaker ALL2 intensity change in H40Y myofibres when compared with WT cells (Fig. 2). In an attempt to identify the origin of such dysfunction (calcium- vs. myosin-related), we overstretched myofibres to mean sarcomere length > 3.60 μm to minimize thin-thick filament overlap [6]. On addition of calcium, the ALL2 intensity change did not differ between WT and H40Y myofibres, proving that the myosin-induced movement is specifically altered and might be a direct consequence of the disrupted myosin function.

## 4 Conclusion

To conclude, our results tend to show that (i) H40Y disrupts the D-loop structure; (ii) normal and mutant actin molecules may form separate filaments, rather than uniform copolymers; and (iii) abnormal monomers/filaments do not undergo any conformational changes during contraction leading to inactive and stiff filaments, preventing proper myosin binding and force generation.

## Transparency document

Transparency Document. Image 1

## Figures and Tables

**Table 1:** Comparison of longitudinal and lateral contacts between H40Y and WT filaments.

		Equilibrated distance (A)	
	Residue ID	H40Y filament	WT filament^a^
Longitudinal contact			
D loop vs. C-term	41 versus 374	12.64 (1.25)	13.09 (1.5)
61 versus 374	23.19 (1.21)	23.29 (1.24)
45 versus 370	14.65 (2.23)	14.85 (2.09)
D loop vs. SD1	45 versus 169	8.62 (1.78)	9.74 (1.74)
61 versus 169	13.27 (1.25)	12.78 (1.4)
D loop vs. SD3	62 versus 288	7.26 (0.53)	7.52 (1.13)
SD4 vs. SD3	205 versus 286	9.36 (0.89)	9.29 (0.53)
241 versus 322	11.08 (1.23)	11.03 (1.76)
Lateral contact			
H-plug vs. C	265 versus 374	19.47 (1.51)	20.13 (1.15)

Standard deviations are included between brackets.

a Data from [17].

**Table 2:** CG representation of subunit geometries reveals major differences between H40Y and WT filaments.

	Parameters	H40Y equilibrated	WT equilibrated^b^
Bond (A)	R1–R2^a^	21.62 (0.35)	22.99 (0.39)
R1–R3	24.90 (0.22)	24.85 (0.28)
R3–R4	24.93 (0.19)	24.97 (0.28)
Angle (°)	R1–R3–R4	74.69 (1.50)	74.42 (1.96)
R2–R1–R3	105.35 (1.90)	102.06 (2.06)
Dihedral (°)	R2–R1–R3–R4	11.14 (2.61)	10.27 (3.41)

Standard deviations are included between brackets.

a Residue sets: R1: 5–33, 80–147, and 334–349; R2: 34–39 and 52–59; R3: 148–179 and 273–333; R4: 180–219 and 252–262.

b Data from [17].
